# Quantitative Assessment of Thyroid Nodules Using Dual-Energy Computed Tomography: Iodine Concentration Measurement and Multiparametric Texture Analysis for Differentiating between Malignant and Benign Lesions

**DOI:** 10.1155/2020/5484671

**Published:** 2020-03-18

**Authors:** Hayato Tomita, Hirofumi Kuno, Kotaro Sekiya, Katharina Otani, Osamu Sakai, Baojun Li, Takashi Hiyama, Keiichi Nomura, Hidefumi Mimura, Tatsushi Kobayashi

**Affiliations:** ^1^Department of Diagnostic Radiology, National Cancer Center Hospital East, Chiba 277-8577, Japan; ^2^Department of Radiology, St. Marianna University School of Medicine, Kawasaki 216-8511, Japan; ^3^AT Innovation Department, Siemens Healthcare K. K., Tokyo 141-8644, Japan; ^4^Department of Radiology, Boston Medical Center, Boston University School of Medicine, Boston 02118, USA

## Abstract

**Results:**

The 34 nodules comprised 14 benign nodules and 20 malignant nodules. Iodine content and Hounsfield unit curve slopes did not differ significantly between benign and malignant thyroid nodules (*P* = 0.480–0.670). However, significant differences in the texture features of monochromatic images were observed between benign and malignant nodules: histogram mean and median, co-occurrence matrix contrast, gray-level gradient matrix (GLGM) skewness, and mean gradients and variance of gradients for GLGM at 80 keV (*P* = 0.014–0.044). The highest AUC was 0.77, for the histogram mean and median of images acquired at 80 keV.

**Conclusions:**

Texture features extracted from monochromatic images using DECT, specifically acquired at high keV, may be a promising diagnostic approach for thyroid nodules. A further large study for incidental thyroid nodules using DECT texture analysis is required to validate our results.

## 1. Introduction

Thyroid nodules are found with increasing frequency, likely due to the widespread use of imaging modalities including ultrasonography (US) and computed tomography (CT). Although more than 95% of the detected nodules are clinically insignificant benign lesions, thyroid nodules are clinically important as they may represent thyroid malignant tumor [1, 2]. Despite an increase in the prevalence of malignant thyroid nodules and invasive examinations, the mortality rate has remained unchanged from 1973; this observation is attributed to overdiagnosis of subclinical thyroid nodules [3, 4]. Therefore, management of incidental thyroid nodules on cross-sectional imaging has been discussed [5–7].

With the recent spread of dual-energy CT (DECT) or spectral CT scanner, dual-energy imaging is widely used in daily practice. DECT offers information on material decomposition, which is acquired from specific changes in the attenuation of each material at two different tube voltages [8–10]. The resulting images provide a spectral attenuation curve plotted within 40 to 140 keV, which allows for characterization of tissues [8–10]. Previously, iodine uptake in thyroid tissues has been known to be associated with thyroid peroxidase (TPO) and sodium iodine symporter (NIS) [11–13]. Li et al. have demonstrated that quantitative ex vivo evaluations using noncontrast DECT have demonstrated a significant difference between benign and malignant thyroid lesions, most likely because cell membranes in normal follicular epithelial cells contain iodine transporters [14]. Therefore, it can be predicted that malignant thyroid nodules would contain less iodine and have lower attenuation on monochromatic images than benign nodules.

With the development of machine learning and the increasing availability of data sets, a quantitative mathematical approach has gained an important role in image diagnosis. Texture analyses based on gray-level changes offer objective features for the quantitative characterization of tissues. Using US and MRI, Acharya et al. [15] and Hao et al. [16] demonstrated significant differences in the texture features of benign and malignant thyroid tumors. We hypothesized that additional quantitative information gained by using multienergy virtual monochromatic image (VMI) data sets could improve the predictive performance of texture analysis.

Hence, the purpose of this study was to investigate the value of quantitative DECT analysis for differentiating between benign and malignant thyroid nodules in vivo, using two approaches: (1) iodine decomposition measurement and slope of CT attenuation and (2) texture analysis of monochromatic images (40 keV, 60 keV, and 80 keV).

## 2. Materials and Methods

This retrospective study was approved by our institutional review board, which waived the need for informed consent from patients.

### 2.1. Subjects

Between April 2015 and March 2016, 52 consecutive patients with thyroid nodules suspected to be malignant underwent noncontrast-enhanced DECT of the neck and US-guided biopsies following our hospital's routine. Among them, 18 patients who had cystic lesions without nodules (*n* = 4) and unavailable DECT data (*n* = 14) were excluded. The remaining 34 patients (9 men and 25 women; age range: 36–84 years; median age: 66 years) were enrolled in this study.

### 2.2. CT Scanning

All patients were scanned using noncontrast 128-row dual-energy CT scanners (SOMATOM Definition Flash; Siemens Healthcare GmBH, Forchheim, Germany). CT scanning settings were as follows: collimation with a z-flying focal spot, 32 × 0.6 mm; tube voltage, 100 and Sn140 kV (where Sn indicates the use of a 0.4 mm tin filter); tube current, 200 effective mAs; gantry rotation time, 0.33 sec; and beam pitch, 0.6. The voltage combination of 100 and Sn140 kV was chosen to minimize noise while maximizing the separation of the X-ray tube's energy spectra [17].

### 2.3. Measurement of Iodine Decomposition and CT Attenuation Slope

**Figure 1:**
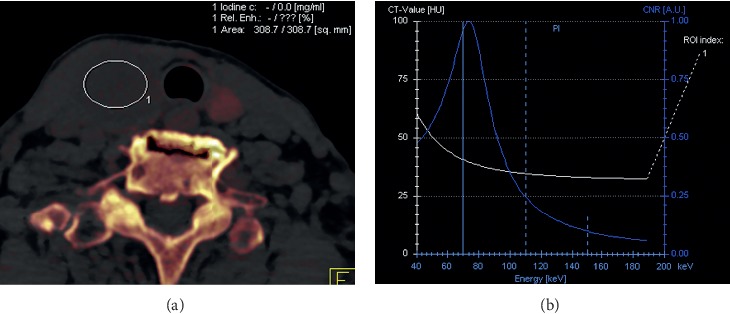
A representative iodine material decomposition image at 120 kVp with the largest region of interest in thyroid nodules selected to exclude cysts and overt calcifications (a) and Hounsfield unit curve slopes at 40 to 190 keV (b).

Scan data were reconstructed as 3 mm thick axial images without overlapping of section intervals. Iodine material decomposition images at 120 kVp and monochromatic images at a range of 40 to 190 keV were reconstructed. We then selected CT slices that demonstrated the maximum diameter of the solid component within nodules of greater than or equal to 10 mm based on the minimum standard value for the fine-needle aspiration in Thyroid Imaging Reporting and Data System by American College of Radiology [18]. Regions of interest (ROIs) were manually drawn for each slice, under consensus between two radiologists (HT and KS, who had 5 and 7 years of experience in head and neck radiology, respectively). ROIs were drawn to include as much of the lesions, whilst avoiding cysts and overt calcifications. The following quantitative parameters were measured: [1] iodine concentration (mg/mL) at 120 kVp and [2] CT attenuation curve slope, plotted from 40 keV to 190 keV (Figure 1).

### 2.4. Image Segmentation and Texture Analysis

**Figure 2:**
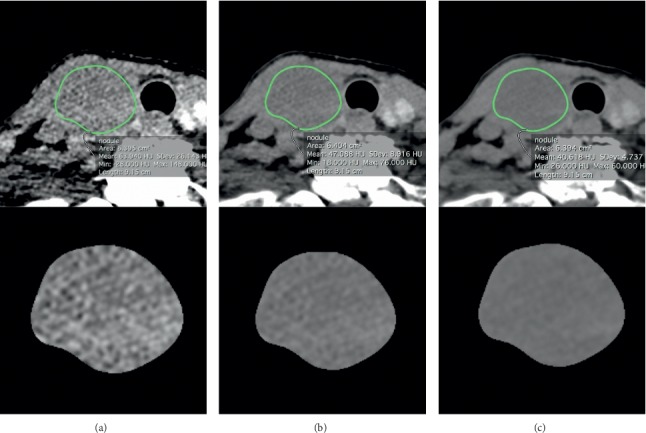
Segmentations of a thyroid nodule on monochromatic images at (a) 40 keV, (b) 60 keV, and (c) 80 keV were performed manually.

Thyroid nodules on 40 keV, 60 keV, and 80 keV monochromatic images were manually delineated by an attending radiologist (∗*blinded*∗, who had 5 years of experience in head and neck radiology) using OsiriX Imaging Software (http://www.osirix-viewer.com). Segmentation was performed on the image set where the tumor was best seen (typically 40 keV VMIs, which has been shown to improve visibility of other head and neck tumors) [19, 20], in conjunction with VMIs at other energies if needed. The solid component was carefully defined to avoid obvious cystic tissue and gross calcification. When severe artifacts were seen within nodules, only artifact-free slices were used for texture analysis. The same ROIs were used to generate VMI data sets for each patient at all 3 energies, taking advantage of the natural coregistration of VMI data sets produced from the same DECT acquisition. A total of 41 features, including 12 histogram features, 5 gray-level co-occurrence matrix (GLCM) features, 11 gray-level run-length (GLRL) features, 4 gray-level gradient matrix (GLGM) features, and 9 Law's features were extracted from each contour, using in-house developed MATLAB-based texture analysis software (MathWorks, Natick, Massachusetts). A detailed summary of all textual features in details measured is shown in [Supplementary-material supplementary-material-1], with mean values presented in Figure 2.

### 2.5. Biopsy Technique and Cytologic Examinations

US-guided biopsies were performed with a 22-gauge needle by experienced head and neck surgeons. Suction was performed after the needle tip was advanced into a solid component of the target lesion. For patients with multiple thyroid nodules, the biopsy was obtained from the largest nodule. Additionally, surgical operations were performed for suspected malignant thyroid nodules that required surgical resection or lymph node resection in clinical practice. Papanicolaou staining was used for cytologic examinations. All specimens were evaluated by an experienced pathologist.

### 2.6. Statistical Analyses

We estimated the sample sizes required to detect differences in iodine content and normalized slopes based on estimates previously reported [14]. Li et al. reported variances in iodine content and normalized slope of 0.4 mg/mL and 0.45 between benign and malignant lesions, respectively, in ex vivo evaluations [14]. In this study, a difference in iodine content of at least 0.4 mg/mL was considered a desirable result [21]. For a two-sided 5% significance level and 80% power, we estimated that a sample size of at least 10 nodules in each group was needed to detect this difference. A difference in normalized slope of at least 0.5 between benign and malignant nodules was desirable. We estimated that a sample size of at least 13 nodules in each group was needed to detect this difference.

Student's *t*-test for independent samples was used to assess differences in iodine content, the normalized slopes of Hounsfield units (HUs), and 41 texture features between benign and malignant nodules at each keV. The mean value of these textural features was calculated per ROI. For each *t*-test performed, equality of variance between the two groups was tested and then the raw *P* value was calculated using either the pooled (equal variance) or Satterthwaite (unequal variance) method. Receiver operating characteristic (ROC) curves were constructed, and the area under the curve (AUC) was measured for each texture feature. *P* values less than or equal to 0.05 were considered statistically significant. All analyses were performed using Stata v. 14.2 statistics software (StataCorp LP, College Station, TX, USA).

## 3. Results

### 3.1. Comparing Iodine Decomposition and CT Attenuation Slope between Benign and Malignant Thyroid Nodules

Histopathological examination of 34 nodules revealed 14 benign and 20 malignant nodules. Among the 20 malignant nodules undergoing FNA, 13 nodules were also confirmed by surgical resection and 2 nodules by lymph node resection. In addition, 15 were diagnosed as papillary, 2 as undifferentiated, 1 as follicular carcinoma, and 2 as lymphoma. The mean ROI area for iodine content and slope measurements was 170 mm^2^, with an interquartile range of 161 mm^2^. The summary statistics for the iodine content and normalized CT attenuation slopes of malignant and benign nodules are shown in Table 1. There was no evidence of a difference in iodine content (0.540 vs 0.437 mg/mL for benign and malignant nodules, respectively; *P*=0.48) or slope (0.836 vs 0.667 for benign and malignant nodules, respectively; *P*=0.67).

### 3.2. Texture Analysis for Differentiating from Benign and Malignant Thyroid Nodules

**Figure 3:**
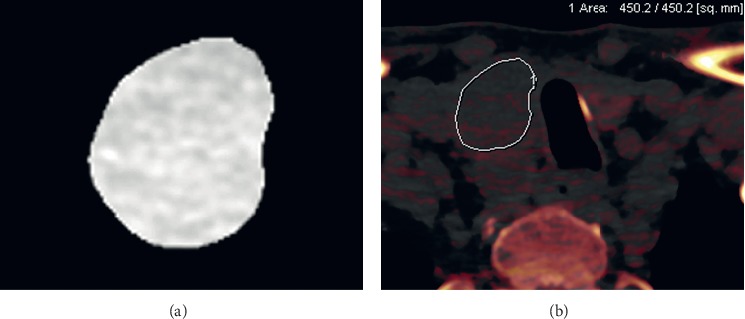
A 62-year-old man with a thyroid papillary carcinoma. On an axial image at 80 keV, a true positive was confirmed using texture features as follows: gray-level co-occurrence matrix (GLCM) mean at 80 keV (1071.9; cut-off value < 1080.9), GLCM median at 80 keV (1071.9; cut-off value < 1080.9) (a). Based on iodine concentration value (0.63 mg/ml) alone, a false negative was determined (b).

**Figure 4:**
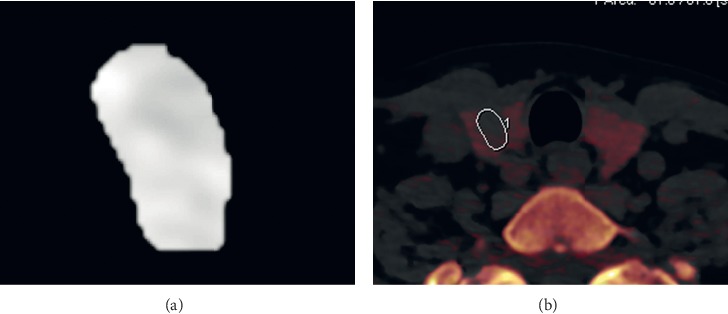
A 59-year-old female with a benign thyroid tumor (follicular adenoma). On an axial image at 80 keV, a true negative was confirmed using texture features as follows: gray-level co-occurrence matrix (GLCM) mean at 80 keV (1082.2; cut-off value > 1080.9), GLCM median at 80 keV (1094.2; cut-off value > 1080.9) (a). Based on iodine concentration value (0.3 mg/ml) alone, a false negative was determined (b).

[Supplementary-material supplementary-material-1] summarizes measurements of 41 texture features at 40, 60, and 80 keV using DECT. Significant differences between benign and malignant thyroid nodules were observed between the histogram mean for 80 keV (*P*=0.019), histogram median at 40 and 80 keV (*P*=0.034 and 0.001, respectively), GLCM contrast for 80 keV (*P*=0.031), GLGM skewness at 40 and 80 keV (*P*=0.030, respectively), and mean gradients (MGR) and gradient variance (VGR) in GLGM at 40, 60, and 80 keV (*P*=0.028–0.044). The AUC, sensitivity, and specificity of selected texture features are shown in Table 2. Based on ROC analysis, the most effective discrimination between benign and malignant nodules was provided by the histogram mean and median at 80 keV, with AUCs of 0.77 for both, sensitivities of 57.1 and 71%, and specificities of 85 and 75%, respectively.  Figure 3 (malignant case) and Figure 4 (benign case) present a representative case in which selected texture features can differentiate between benign and malignant thyroid nodules at high keV on DECT, whereas material decomposition data were unable to discriminate between them.

## 4. Discussion

Six texture features on three monochromatic images met the desired performance standard for distinguishing between benign and malignant thyroid nodules in vivo. Notably, DECT material decomposition and CT attenuation slopes did not show significant differences. These results suggest that this approach to texture analysis using extracted monochromatic images may be a valuable imaging tool for diagnosing thyroid tumors using noncontrast CT. Ultrasound (US) has been used to assess thyroid nodules, and US-guided biopsies are a standard procedure in the establishment of the diagnosis of benign and malignant thyroid nodules. However, US is dependent on the operator's technique and does not clearly visualize the mediastinal lesions. Furthermore, thyroid nodules are often unintentionally identified by CT. The texture analysis is a postprocessing technique that assesses the tissue characteristics of the tumors without exposing the patient to additional radiation. If a thyroid nodule is discovered on DECT in daily clinical practice, we believe that texture analysis using extracted monochromatic images can provide additional information for further examination, including US.

After the introduction of high-resolution CT scanners, previous studies have demonstrated that imaging features including punctuate calcification, nodule size, and irregularity could help detect malignant thyroid nodules [22–24]. DECT has spread widely at a variety of institutions and has been used in daily practice. Li et al. demonstrated that there was a potential to diagnose ex vivo thyroid nodules using DECT by detecting the amount of iodine and changes in HU attenuation slopes in tissues [14]. Previous studies have demonstrated that the expressions of TPO mRNA and NIS in malignant thyroid nodules were less than in benign lesions [11–13]. In the process of producing thyroid hormones, TPO incorporates inorganic iodine into organic iodine in the follicular epithelial cells. NIS expression on the basolateral membrane of the thyroid follicular cells leads to iodine accumulation. Malignant thyroid nodules have comparatively less iodine, due to the decreased expression of TPO mRNA and NIS, than benign thyroid nodules. However, Ringel et al. also demonstrated that there was no significant difference in the NIS expression between malignant and benign thyroid nodules [12]. Additionally, we found no significant differences between benign and malignant thyroid nodules using material decomposition and attenuation slopes for monochromatic images obtained in vivo. Although it is difficult to determine the exact reason for this difference, we propose several possible causes. First, other tissues within the scan range, including fat, muscle, and bone, would affect the attenuation of X-rays at different energies and make it difficult to detect low iodine concentrations. Li et al. demonstrated that the ex vivo iodine concentrations within goiters, follicular adenomas, and malignant groups were 0.42 ± 0.37 mg/mL, 0.51 ± 0.42 mg/mL, and 0.41 ± 0.2 mg/mL, respectively [14]. In addition, Ringel et al. also demonstrated that there was no significant difference in NIS expression between malignant and benign thyroid nodules [12]. These differences might be difficult to detect in the presence of more complex tissue components. Second, the dual source scanner used in this study may have more significant problems with cross-scatter than the fast kilovoltage-switching dual-energy scanner used in the previous study. Cross-scatter results in noise due to the collision of two X-rays emitted from different sources at a perpendicular angle to each other [25, 26].

Measures of texture analysis such as smoothness and regularity in pixels represent both distributions and relationships in gray-level patterns [27]. Texture analysis has been applied to the field of oncology to analyze predictors, treatment responses, and histologic classification [28–32]. Heterogeneity in the malignant tissues is recognized to reflect cellularity, necrosis, and angiogenesis, which are associated with disorders of attenuation [33]. Previous studies demonstrated that texture analysis of images obtained from US and MRI is useful for distinguishing between benign thyroid nodules and malignancies with less-uniform texture parameters, classifiers, and applications [15, 16, 34–36]. Since thyroid nodules are often detected by CT scan before performing further US, with texture analysis using DECT, unnecessary further examinations of the thyroid lesions can be potentially prevented.

Al Ajmi et al. previously described first-order statistics for texture features that could be used to classify benign parotid tumors and predict the effectiveness of induction chemotherapy in patients with head and neck carcinoma, respectively [32, 37]. In this study, the histogram mean and median at 80 keV were higher for benign than malignant lesions. Furthermore, these factors resulted in the best AUCs for differentiating between the two. GLGM MGR and VGR, which are indicative of gray-level distortions, were lower for benign lesions. These findings would suggest that, in 80 keV images, benign thyroid lesions have less heterogeneity than malignant thyroid lesions. At higher keV, tumor and soft tissue attenuations are inferior to those of lower keV on monochromatic images; however, image noise decreases at higher keV [20, 38]. CT imaging of the thyroid is expected to be more susceptible to beam hardening artifacts and image noise, due to the proximity of the clavicles and shoulders. Therefore, a decreased standard deviation at higher keV might have a small influence on mean texture features between benign and malignant thyroid nodules, compared with imaging at lower keV.

Despite these findings, some limitations to the present study need to be considered. In our retrospective study, our investigations of texture analysis on monochromatic images were performed in a small number of patients with a high prevalence of malignant thyroid nodules rather than in patients with incidental malignant thyroid nodules. This may lead to overfitting results. Second, this is a preliminary first demonstration regarding the potential use of DECT texture analysis based on a relatively small number of patients with thyroid nodule; certainly, future investigation both using a larger number of patients and comparing DECT with US is required. Third, benign thyroid nodules were followed for at least for 1 year clinically; therefore, their development into malignant lesions may not have been detected within the study time frame. Lastly, obvious cystic changes in lesions were excluded from quantitative measurements because it was thought that cystic tissue might affect the accurate analysis of solid components. Therefore, a potentially confounding effect of interreader error would be a source of bias. These problems could be solved in future studies with a larger sample size.

## 5. Conclusions

In conclusion, this study investigated the imaging characteristics of benign and malignant thyroid nodules in vivo using quantitative DECT. If a thyroid nodule is discovered on DECT in daily clinical practice, texture analysis of monochromatic images acquired at high keV may be a more promising diagnostic approach for thyroid nodules than DECT material decomposition and CT attenuation slopes.

## Figures and Tables

**Table 1:** Iodine content and slope of benign and malignant thyroid nodules.

	Benign lesions (*n* = 14)		Malignant lesions (*n* = 20)		AUC
	Mean	SD	Mean	SD	
Iodine content (mg/mL)	0.54	0.849	0.437	0.542	0.564
Slope	0.836	0.924	0.667	0.609	0.571

SD = standard deviation; AUC = area under receiver operating characteristic curve.

**Table 2:** Diagnostic performance of selected texture features to differentiate benign thyroid nodules from malignant nodules.

Texture parameter	Benign lesions (*n* = 14)		Malignant lesions (*n* = 20)		*P* value	Cut-off	Sensitivity (%)	Specificity (%)	AUC
	Mean	SD	Mean	SD					
Mean in histogram									
40 keV	1124.8	39.6	1091.3	52.3	0.052	N/A	N/A	N/A	0.682
60 keV	1091.9	26.2	1059.8	94.5	0.163	N/A	N/A	N/A	0.664
80 keV	1085.7	18.5	1071.9	9.4	0.019^*∗*^	<1080.9	57.1	85.0	0.771^¶^

Median in histogram									
40 keV	1124.8	39.2	1099.7	27.1	0.034^*∗*^	<1112.0	64.3	85.0	0.686
60 keV	1092.8	26.0	1072.6	39.4	0.103	N/A	N/A	N/A	0.679
80 keV	1086.7	18.6	1071.9	9.5	0.014^*∗*^	<1080.9	71.0	75.0	0.771^¶^

Contrast in GLCM									
40 keV	23.6	18.9	18.0	12.5	0.309	N/A	N/A	N/A	0.629
60 keV	21.3	20.1	17.2	27.1	0.635	N/A	N/A	N/A	0.629
80 keV	18.5	15.3	8.4	4.3	0.031^*∗*^	<15.12	50.0	85.0	0.693

Skewness in GLGM									
40 keV	36.3	12.3	28.6	7.6	0.030^*∗*^	<37.00	50.0	85.0	0.696
60 keV	36.0	12.2	28.8	7.4	0.064	N/A	N/A	N/A	0.671
80 keV	36.4	12.0	28.8	7.4	0.030^*∗*^	<29.49	78.6	65.0	0.696

MGR in GLGM									
40 keV	5.792	3.559	9.340	5.805	0.035^*∗*^	>3.301	95.0	35.7	0.689
60 keV	5.041	2.880	7.993	4.479	0.038^*∗*^	>3.692	85.0	50.0	0.693
80 keV	4.850	2.696	7.615	4.199	0.038^*∗*^	>3.639	85.0	50.0	0.704

VGR in GLGM									
40 keV	19974.2	10745.3	30478.1	16424.1	0.044^*∗*^	>15036	85.0	42.4	0.689
60 keV	18824.2	10530.9	28949.7	15235.9	0.039^*∗*^	>12098	95.0	35.7	0.696
80 keV	17957.5	10154.8	28800.9	15431.2	0.028^*∗*^	>14288	85.0	50.0	0.721

GLCM = gray-level co-occurrence matrix; GLGM = gray-level gradient matrix; MGR = mean gradients; VGR = a variance of gradients; AUC = area under receiver operating characteristic curve; ^*∗*^significant differences are defined as *P* < 0.05; ^¶^highest AUC among 41 texture features.

## Data Availability

The clinical data used to support the findings of this study are included within the article.

## Supplementary Materials

Supplementary Materials Table E1: comparison of texture parameters between benign and malignant thyroid nodules at 40 keV. Table E2: comparison of texture parameters between benign and malignant thyroid nodules at 60 keV. Table E3: comparison of texture parameters between benign and malignant thyroid nodules at 80 keV.

## References

[1] Maruchi N., Furihata R., Makiuchi M. (1971). Population surveys on the prevalence of thyroid cancer in a non-endemic region, Nagano, Japan. *International Journal of Cancer*.

[2] Suehiro F. (2006). Thyroid cancer detected by mass screening over a period of 16 years at a health care center in Japan. *Surgery Today*.

[3] Davies L., Welch H. G. (2006). Increasing incidence of thyroid cancer in the United States, 1973–2002. *JAMA*.

[4] Hoang J. K., Choudhury K. R., Eastwood J. D. (2014). An exponential growth in incidence of thyroid cancer: trends and impact of CT imaging. *American Journal of Neuroradiology*.

[5] Tanpitukpongse T. P., Grady A. T., Sosa J. A., Eastwood J. D., Choudhury K. R., Hoang J. K. (2015). Incidental thyroid nodules on CT or MRI: discordance between what we report and what receives workup. *American Journal of Roentgenology*.

[6] Hoang J. K., Middleton W. D., Farjat A. E. (2018). Reduction in thyroid nodule biopsies and improved accuracy with American College of Radiology Thyroid Imaging Reporting and Data System. *Radiology*.

[7] Hoang J. K., Langer J. E., Middleton W. D. (2015). Managing incidental thyroid nodules detected on imaging: white paper of the ACR incidental thyroid findings committee. *Journal of the American College of Radiology*.

[8] Forghani R., Srinivasan A., Forghani B. (2017). Advanced tissue characterization and texture analysis using dual-energy computed tomography. *Neuroimaging Clinics of North America*.

[9] McCollough C. H., Leng S., Yu L., Fletcher J. G. (2015). Dual- and multi-energy CT: principles, technical approaches, and clinical applications. *Radiology*.

[10] Johnson T. R. C. (2012). Dual-energy CT: general principles. *American Journal of Roentgenology*.

[11] Lazar V., Bidart J. M., Caillou B. (1999). Expression of the Na+/I- symporter gene in human thyroid tumors: a comparison study with other thyroid-specific genes. *Journal of Clinical Endocrinology & Metabolism*.

[12] Ringel M. D., Anderson J., Souza S. L. (2001). Expression of the sodium iodide symporter and thyroglobulin genes are reduced in papillary thyroid cancer. *Modern Pathology*.

[13] Sodré A. K. M. B., Rubio I. G. S., Galrão A. L. R. (2008). Association of low sodium-iodide symporter messenger ribonucleic acid expression in malignant thyroid nodules with increased intracellular protein staining. *The Journal of Clinical Endocrinology & Metabolism*.

[14] Li M., Zheng X., Li J. (2012). Dual-energy computed tomography imaging of thyroid nodule specimens. *Investigative Radiology*.

[15] Acharya U. R., Vinitha Sree S., Krishnan M. M., Molinari F., Garberoglio R., Suri J. S. (2012). Non-invasive automated 3D thyroid lesion classification in ultrasound: a class of ThyroScan systems. *Ultrasonics*.

[16] Hao Y., Pan C., Chen W., Li T., Zhu W., Qi J. (2016). Differentiation between malignant and benign thyroid nodules and stratification of papillary thyroid cancer with aggressive histological features: whole-lesion diffusion-weighted imaging histogram analysis. *Journal of Magnetic Resonance Imaging*.

[17] Kuno H., Onaya H., Iwata R. (2012). Evaluation of cartilage invasion by laryngeal and hypopharyngeal squamous cell carcinoma with dual-energy CT. *Radiology*.

[18] Haugen B. R., Alexander E. K., Bible K. C. (2016). 2015 American thyroid association management guidelines for adult patients with thyroid nodules and differentiated thyroid cancer: the american thyroid association guidelines task force on thyroid nodules and differentiated thyroid cancer. *Thyroid*.

[19] Lam S., Gupta R., Levental M., Yu E., Curtin H. D., Forghani R. (2015). Optimal virtual monochromatic images for evaluation of normal tissues and head and neck cancer using dual-energy CT. *American Journal of Neuroradiology*.

[20] Forghani R., Kelly H., Yu E. (2017). Low-energy virtual monochromatic dual-energy computed tomography images for the evaluation of head and neck squamous cell carcinoma. *Journal of Computer Assisted Tomography*.

[21] Gupta K., Attri J., Singh A., Kaur H., Kaur G. (2016). Basic concepts for sample size calculation: critical step for any clinical trials!. *Saudi Journal of Anaesthesia*.

[22] Ishigaki S., Shimamoto K., Satake H. (2004). Multi-slice CT of thyroid nodules: comparison with ultrasonography. *Radiation Medicine*.

[23] Shetty S. K., Maher M. M., Hahn P. F., Halpern E. F., Aquino S. L. (2006). Significance of incidental thyroid lesions detected on CT: correlation among CT, sonography, and pathology. *American Journal of Roentgenology*.

[24] Kim D. W., Jung S. J., Baek H. J. (2015). Computed tomography features of benign and malignant solid thyroid nodules. *Acta Radiologica*.

[25] Forghani R., De Man B., Gupta R. (2017). Dual-energy computed tomography. *Neuroimaging Clinics of North America*.

[26] Forghani R., De Man B., Gupta R. (2017). Dual-energy computed tomography. *Neuroimaging Clinics of North America*.

[27] Acharya U. R., Faust O., Sree S. V., Molinari F., Garberoglio R., Suri J. S. (2011). Cost-effective and non-invasive automated benign & malignant thyroid lesion classification in 3D contrast-enhanced ultrasound using combination of wavelets and textures: a class of ThyroScan algorithms. *Technology in Cancer Research & Treatment*.

[28] Buch K., Fujita A., Li B., Kawashima Y., Qureshi M. M., Sakai O. (2015). Using texture analysis to determine human papillomavirus status of oropharyngeal squamous cell carcinomas on CT. *American Journal of Neuroradiology*.

[29] Joseph G. B., Baum T., Carballido-Gamio J. (2011). Texture analysis of cartilage T2 maps: individuals with risk factors for OA have higher and more heterogeneous knee cartilage MR T2 compared to normal controls - data from the osteoarthritis initiative. *Arthritis Research & Therapy*.

[30] Yu H., Buch K., Li B. (2015). Utility of texture analysis for quantifying hepatic fibrosis on proton density MRI. *Journal of Magnetic Resonance Imaging*.

[31] Kuno H., Qureshi M. M., Chapman M. N. (2017). CT texture analysis potentially predicts local failure in head and neck squamous cell carcinoma treated with chemoradiotherapy. *American Journal of Neuroradiology*.

[32] Al Ajmi E., Forghani B., Reinhold C., Bayat M., Forghani R. (2018). Spectral multi-energy CT texture analysis with machine learning for tissue classification: an investigation using classification of benign parotid tumours as a testing paradigm. *European Radiology*.

[33] Bayanati H., Thornhill R. E., Souza C. A. (2015). Quantitative CT texture and shape analysis: can it differentiate benign and malignant mediastinal lymph nodes in patients with primary lung cancer?. *European Radiology*.

[34] Tsantis S., Dimitropoulos N., Cavouras D., Nikiforidis G. (2009). Morphological and wavelet features towards sonographic thyroid nodules evaluation. *Computerized Medical Imaging and Graphics*.

[35] Schob S., Meyer H., Dieckow J. (2017). Histogram analysis of diffusion weighted imaging at 3T is useful for prediction of lymphatic metastatic spread, proliferative activity, and cellularity in thyroid cancer. *International Journal of Molecular Sciences*.

[36] Kim S.-Y., Kim E.-K., Moon H. J., Yoon J. H., Kwak J. Y. (2015). Application of texture analysis in the differential diagnosis of benign and malignant thyroid nodules: comparison with gray-scale ultrasound and elastography. *American Journal of Roentgenology*.

[37] Zhang H., Graham C. M., Elci O. (2013). Locally advanced squamous cell carcinoma of the head and neck: CT texture and histogram analysis allow independent prediction of overall survival in patients treated with induction chemotherapy. *Radiology*.

[38] Wichmann J. L., Nöske E.-M., Kraft J. (2014). Virtual monoenergetic dual-energy computed tomography. *Investigative Radiology*.

