# Supplementary material for: Quantitative Assessment of Thyroid Nodules Using Dual-Energy Computed Tomography: Iodine Concentration Measurement and Multiparametric Texture Analysis for Differentiating between Malignant and Benign Lesions
Source: Int J Endocrinol. 2020 Mar 18;2020:5484671. doi: 10.1155/2020/5484671 (PMC7104273; doi:10.1155/2020/5484671)
Supplement: Supplementary Materials — Table E1: comparison of texture parameters between benign and malignant thyroid nodules at 40 keV. Table E2: comparison of texture parameters between benign and malignant thyroid nodules at 60 keV. Table E3: comparison of texture parameters between benign and malignant thyroid nodules at 80 keV. [file 5484671.f1.docx]

**SUPPLEMENTARY MATERIALS**

**Appendix 1**

1. **Histogram features**

Histogram features consist of simple statistics that are associated with pixel values in images with the spatial patterns of pixel values not included. Mean, median, standard deviation (SD), entropy, range, geometric mean, harmonic mean, interquartile range (IQR), fourth moment, standard deviation in a 5-pixel neighborhood (SD5), and standard deviation in a 9-pixel neighborhood (SD 9) were calculated.

1. **Gray level co-occurrence matrix features**

The gray level co-occurrence matrix (GLCM) is a symmetric matrix with rows and columns from 0 to Ng, where Ng shows the number of gray levels. The co-occurrence matrix is calculated based on the distance between a specific pair of pixels in an image and the direction (0°, 45°, 90°, 135°) {{343 Buch,K. 2015; 368 Kuno,H. 2017;}}. This study used the following features proposed by Haralick et al. {{364 Haralick R, Shanmugam K, Dinstein I. 1973;}}:

Contrast = $\sum_{i,j} {|i-j|}^{2}p$(*i*, *j*)

Correlation = $\sum_{i,j} \frac{(i-\mu_{i})(j-\mu_{j})p(i, j)}{\sigma_{i}\sigma_{j}}$

Angular Second Moment (ASM) = $\sum_{i,j} p{(i, j)}^{2}$

Homogeneity = $\sum_{i,j} \frac{p(i,j)}{1+|i-j|}$

Entropy = $\sum_{i,j} \ln\left( p(i,j \right))p(i,j)$

where *p(i,j)* represents *(i,j)* value of the GLCM.

1. **Gray level run-length features**

The Gray level run-length (GLRL) matrix is the number of runs with pixels of gray levels i and run length j. Run length j is the number of consecutive pixels of the same gray level value for a specific direction. In this study, we used the following features; short run emphasis (SRE), long run emphasis (LRE), gray level non-uniformity (GLN), run-length nonuniformity (RLN), run percentage (RP), low gray level run emphasis (LGRE), high gray level run emphasis (HGRE), short run low gray level emphasis (SRLGE), short run high gray level emphasis (SRHGE), long run low gray level emphasis (LRLGE), and long run high gray level emphasis (LRHGE) {{368 Kuno,H. 2017;}}.

SRE = $\frac{1}{n_{r}}\sum_{i,j} \frac{p(i,j)}{j^{2}}$

LRE = $\frac{1}{n_{r}}\sum_{i,j} p(i,j)j^{2}$

GLN = $\frac{1}{n_{r}}\sum_{i} \left( \sum_{j} p(i,j) \right)^{2}$

RLN = $\frac{1}{n_{r}}\sum_{j} \left( \sum_{i} p(i,j) \right)^{2}$

RP = $\frac{n_{r}}{n_{p}}$

LGRE = $\frac{1}{n_{r}}\sum_{i,j} \frac{p(i,j)}{i^{2}}$

HGRE = $\frac{1}{n_{r}}\sum_{i,j} p(i,j)i^{2}$

SRLGE = $\frac{1}{n_{r}}\sum_{i,j} \frac{p(i,j)}{i^{2}j^{2}}$

SRHGE = $\frac{1}{n_{r}}\sum_{i,j} \frac{p(i,j)i^{2}}{j^{2}}$

LRLGE = $\frac{1}{n_{r}}\sum_{i,j} \frac{p(i,j)j^{2}}{i^{2}}$

LRHGE = $\sum_{i,j} p(i,j){i^{2}j}^{2}$

1. **Gray level gradient matrix features**

The grey level gradient matrix refers to relationships between gray level values and gradient values of pixels in a segmentation, including the mean, variance, skewness, and kurtosis.

1. **Laws features**

Laws features were produced from filter masks of 5 x 5 convolution, generated from “vector level”, “edge”, “ripple”, “wave”, and “spot” in images, as described previously {{367 Laws KI. 1980; 368 Kuno,H. 2017;}}. The final 9 filter masks (L1-L9) were formed by combining certain symmetric pairs {{365 Li,B. 2017; 368 Kuno,H. 2017;}}.

**Appendix 2**

**Table E1:** Comparison of texture parameters between benign and malignant thyroid nodules at 40 keV

|  | **40keV** | | | | | | |
| --- | --- | --- | --- | --- | --- | --- | --- |
| Texture parameter | Benign | |  | Malignant | |  | P-value |
|  | mean | SD |  | mean | SD |  |  |
| Histogram |  |  |  |  |  |  |  |
| mean | 1124.8 | 39.6 |  | 1091.3 | 52.3 |  | 0.052 |
| median | 1124.8 | 39.2 |  | 1099.7 | 27.1 |  | 0.034* |
| std | 35.8 | 11.4 |  | 57.2 | 89.3 |  | 0.300 |
| entropy | 6.657 | 0.504 |  | 6.659 | 0.485 |  | 0.995 |
| 2D std | 13.76 | 4.53 |  | 14.04 | 4.78 |  | 0.862 |
| range | 40.5 | 13.2 |  | 41.1 | 13.7 |  | 0.886 |
| geometric mean | 1124.1 | 40.0 |  | 1067.0 | 152.6 |  | 0.124 |
| harmonic mean | 1123.42 | 40.19 |  | 1046.71 | 239.02 |  | 0.174 |
| iqr | 48.7 | 15.8 |  | 65.5 | 74.8 |  | 0.336 |
| 4th moment | 1.17E+07 | 1.32E+07 |  | 4.71E+09 | 2.09E+10 |  | 0.329 |
| std 5 | 20.0 | 6.3 |  | 19.9 | 6.8 |  | 0.967 |
| std 9 | 25.9 | 2.0 |  | 25.8 | 2.0 |  | 0.971 |
| GLCM |  |  |  |  |  |  |  |
| entropy | 1.376 | 0.599 |  | 1.617 | 0.575 |  | 0.247 |
| contrast | 23.6 | 18.9 |  | 18.0 | 12.5 |  | 0.309 |
| correlation | 0.850 | 0.085 |  | 0.842 | 0.106 |  | 0.818 |
| energy | 0.005 | 0.003 |  | 0.011 | 0.025 |  | 0.337 |
| homogeneity | 0.369 | 0.100 |  | 0.410 | 0.088 |  | 0.218 |
| GLRL |  |  |  |  |  |  |  |
| SRE | 0.017 | 0.006 |  | 0.022 | 0.028 |  | 0.465 |
| LRE | 0.017 | 0.006 |  | 0.022 | 0.027 |  | 0.495 |
| GLN | 0.017 | 0.006 |  | 0.021 | 0.231 |  | 0.494 |
| RLN | 0.017 | 0.006 |  | 0.021 | 0.026 |  | 0.482 |
| RP | 224.8 | 29.0 |  | 228.9 | 57.7 |  | 0.786 |
| LGRE | 223.5 | 28.3 |  | 228.7 | 59.0 |  | 0.739 |
| HGRE | 224.2 | 28.9 |  | 229.1 | 59.5 |  | 0.755 |
| SRLGE | 224.3 | 29.1 |  | 229.2 | 59.7 |  | 0.753 |
| SRHGE | 1320.4 | 1612.3 |  | 1954.5 | 1866.8 |  | 0.299 |
| LRLGE | 2059.1 | 2763.0 |  | 2702.2 | 2529.7 |  | 0.492 |
| LRHGE | 1700.9 | 2053.9 |  | 2264.8 | 2057.4 |  | 0.438 |
| Law's features |  |  |  |  |  |  |  |
| L1 | 618477.3 | 417187.1 |  | 437533.2 | 160694.1 |  | 0.175 |
| L2 | 131278.8 | 135009.9 |  | 63966.3 | 34288.2 |  | 0.117 |
| L3 | 28984.1 | 18412.2 |  | 21260.5 | 7497.6 |  | 0.190 |
| L4 | 175334.6 | 121952.2 |  | 120212.6 | 42479.3 |  | 0.156 |
| L5 | 35408.4 | 35347.6 |  | 18157.4 | 13040.5 |  | 0.130 |
| L6 | 24164.7 | 23545.5 |  | 13208.5 | 9116.6 |  | 0.142 |
| L7 | 13920.7 | 10588.7 |  | 9246.4 | 4149.9 |  | 0.169 |
| L8 | 57050.3 | 56376.1 |  | 30655.9 | 22191.5 |  | 0.146 |
| L9 | 73871.3 | 40015.3 |  | 58259.1 | 4014.2 |  | 0.223 |
| GLGM |  |  |  |  |  |  |  |
| MGR | 5.792 | 3.559 |  | 9.340 | 5.805 |  | 0.035* |
| VGR | 19974.2 | 10745.3 |  | 30478.1 | 16424.1 |  | 0.044* |
| skewness | 36.3 | 12.3 |  | 28.6 | 7.6 |  | 0.030* |
| kurtosis | 1539.1 | 1090.9 |  | 937.6 | 469.1 |  | 0.070 |

* = Significant differences are defined as *p* < 0.05

*Definition of abbreviations:* GLCM = gray level co-occurrence matrix; GLRL = grya-level run length; SRE = short-run emphasis; LRE = long-run emphasis; GLN = gray-level nonuniformity; RLN = run-length nonuniformity; RP = run percentage; LGRE = low gray-level run emphasis; HGRE = high gray-level run emphasis; SLRGE = short-run low gray-level emphasis; SLHGE = short-run high gray-level emphasis; LRLGE = long-run low gray-level emphasis; LRHGE = short-run high gray-level emphasis; GLGM = Gray level gradient matrix; MGR = mean gradients; VGR = a variance of gradients; SD = standard diviation.

**Table E2:** Comparison of texture parameters between benign and malignant thyroid nodules at 60 keV

|  | **60keV** | | | | | |
| --- | --- | --- | --- | --- | --- | --- |
| Texture parameter | Benign | |  | Malignant | | P-value |
|  | mean | SD |  | mean | SD |  |
| Histogram |  |  |  |  |  |  |
| mean | 1091.9 | 26.2 |  | 1059.8 | 94.5 | 0.163 |
| median | 1092.8 | 26.0 |  | 1072.6 | 39.4 | 0.103 |
| std | 29.1 | 63.9 |  | 51.4 | 116.9 | 0.479 |
| entropy | 5.275 | 0.561 |  | 5.584 | 0.800 | 0.222 |
| 2D std | 4.47 | 2.44 |  | 9.62 | 20.78 | 0.286 |
| range | 13.1 | 7.1 |  | 28.7 | 63.4 | 0.290 |
| geometric mean | 1078.4 | 58.9 |  | 1028.8 | 193.7 | 0.293 |
| harmonic mean | 1030.6 | 233.1 |  | 985.1 | 296.4 | 0.636 |
| iqr | 16.3 | 4.6 |  | 73.6 | 230.3 | 0.280 |
| 4th moment | 3.14E+09 | 1.17E+10 |  | 6.39E+09 | 2.05E+10 | 0.608 |
| std 5 | 6.2 | 2.5 |  | 11.4 | 19.0 | 0.237 |
| std 9 | 7.8 | 2.8 |  | 15.7 | 27.6 | 0.217 |
| GLCM |  |  |  |  |  |  |
| entropy | 1.2 | 0.6 |  | 1.3 | 0.5 | 0.340 |
| contrast | 21.30 | 20.13 |  | 17.20 | 27.18 | 0.635 |
| correlation | 0.9 | 0.1 |  | 0.8 | 0.2 | 0.860 |
| energy | 0.030 | 0.084 |  | 0.028 | 0.084 | 0.954 |
| homogeneity | 0.442 | 0.162 |  | 0.466 | 0.112 | 0.614 |
| GLRL |  |  |  |  |  |  |
| SRE | 0.021 | 0.011 |  | 0.296 | 0.039 | 0.369 |
| LRE | 0.020 | 0.009 |  | 0.296 | 0.040 | 0.330 |
| GLN | 0.021 | 0.011 |  | 0.292 | 0.039 | 0.377 |
| RLN | 0.021 | 0.010 |  | 0.030 | 0.041 | 0.353 |
| RP | 240.1 | 63.7 |  | 240.3 | 76.2 | 0.985 |
| LGRE | 240.0 | 65.7 |  | 240.4 | 81.1 | 0.990 |
| HGRE | 239.1 | 62.3 |  | 240.3 | 80.0 | 0.963 |
| SRLGE | 240.1 | 65.7 |  | 240.7 | 80.4 | 0.981 |
| SRHGE | 963.8 | 830.3 |  | 1352.8 | 1270.4 | 0.323 |
| LRLGE | 1572.1 | 1632.7 |  | 2026.1 | 1887.2 | 0.472 |
| LRHGE | 1236.6 | 1097.8 |  | 1626.9 | 1428.2 | 0.397 |
| Law's features |  |  |  |  |  |  |
| L1 | 421392.7 | 400538.8 |  | 319997.8 | 197043.7 | 0.429 |
| L2 | 85675.2 | 119481.1 |  | 43152.9 | 33145.0 | 0.252 |
| L3 | 15627.1 | 15247.7 |  | 11520.3 | 7876.3 | 0.403 |
| L4 | 105421.8 | 11-598.4 |  | 73537.9 | 46489.1 | 0.361 |
| L5 | 21682.7 | 25912.7 |  | 12509.6 | 11119.4 | 0.267 |
| L6 | 16101.0 | 20466.8 |  | 9172.7 | 8062.8 | 0.284 |
| L7 | 8324.6 | 9173.3 |  | 5573.7 | 4334.8 | 0.349 |
| L8 | 37741.3 | 48575.3 |  | 21274.3 | 18444.6 | 0.282 |
| L9 | 41422.1 | 35308.1 |  | 34021.4 | 19886.8 | 0.519 |
| GLGM |  |  |  |  |  |  |
| MGR | 5.041 | 2.880 |  | 7.993 | 4.479 | 0.038* |
| VGR | 18824.2 | 10530.9 |  | 28949.7 | 15235.9 | 0.039* |
| skewness | 36.0 | 12.2 |  | 28.8 | 7.4 | 0.064 |
| kurtosis | 1508.0 | 1094.3 |  | 944.8 | 470.4 | 0.088 |

* = Significant differences are defined as *p* < 0.05

*Definition of abbreviations:* GLCM = gray level co-occurrence matrix; GLRL = grya-level run length; SRE = short-run emphasis; LRE = long-run emphasis; GLN = gray-level nonuniformity; RLN = run-length nonuniformity; RP = run percentage; LGRE = low gray-level run emphasis; HGRE = high gray-level run emphasis; SLRGE = short-run low gray-level emphasis; SLHGE = short-run high gray-level emphasis; LRLGE = long-run low gray-level emphasis; LRHGE = short-run high gray-level emphasis; GLGM = Gray level gradient matrix; MGR = mean gradients; VGR = a variance of gradients; SD = standard diviation.

**Table E3:** Comparison of texture parameters between benign and malignant thyroid nodules at 80 keV

|  | **80keV** | | | | | |
| --- | --- | --- | --- | --- | --- | --- |
| Texture parameter | Benign | |  | Malignant | | P-value |
|  | mean | SD |  | mean | SD |  |
| Histogram |  |  |  |  |  |  |
| mean | 1085.7 | 18.5 |  | 1071.9 | 9.4 | 0.019* |
| median | 1086.7 | 18.6 |  | 1071.9 | 9.5 | 0.014* |
| std | 29.4 | 65.0 |  | 8.4 | 2.6 | 0.248 |
| entropy | 4.940 | 0.971 |  | 4.871 | 0.459 | 0.809 |
| 2D std | 4.6 | 6.38 |  | 2.16 | 0.56 | 0.169 |
| range | 13.5 | 18.6 |  | 6.3 | 1.6 | 0.167 |
| geometric mean | 1072.2 | 54.2 |  | 1071.9 | 9.4 | 0.984 |
| harmonic mean | 1024.2 | 230.5 |  | 1071.9 | 9.371 | 0.454 |
| iqr | 16.6 | 17.5 |  | 10.9 | 3.941 | 0.247 |
| 4th moment | 3.14E+09 | 1.17E+10 |  | 7.95E+04 | 1.25E+05 | 0.335 |
| std 5 | 6.4 | 8.9 |  | 3.1 | 0.8 | 0.187 |
| std 9 | 8.27 | 11.05 |  | 4.30 | 1.16 | 0.203 |
| GLCM |  |  |  |  |  |  |
| entropy | 0.997 | 0.484 |  | 1.195 | 0.398 | 0.199 |
| contrast | 18.5 | 15.3 |  | 8.4 | 4.3 | 0.031* |
| correlation | 0.869 | 0.105 |  | 0.918 | 0.039 | 0.120 |
| energy | 0.032 | 0.083 |  | 0.012 | 0.007 | 0.380 |
| homogeneity | 0.451 | 0.136 |  | 0.488 | 0.060 | 0.426 |
| GLRL |  |  |  |  |  |  |
| SRE | 0.021 | 0.013 |  | 0.019 | 0.012 | 0.608 |
| LRE | 0.021 | 0.012 |  | 0.019 | 0.013 | 0.737 |
| GLN | 0.021 | 0.013 |  | 0.019 | 0.012 | 0.663 |
| RLN | 0.021 | 0.011 |  | 0.019 | 0.013 | 0.761 |
| RP | 253.7 | 57.1 |  | 227.8 | 47.8 | 0.162 |
| LGRE | 253.9 | 59.9 |  | 226.3 | 48.4 | 0.140 |
| HGRE | 253.5 | 55.8 |  | 226.4 | 47.5 | 0.138 |
| SRLGE | 254.2 | 59.0 |  | 226.5 | 48.3 | 0.142 |
| SRHGE | 1101.0 | 1481.7 |  | 1205.9 | 1148.3 | 0.818 |
| LRLGE | 1632.8 | 2116.2 |  | 1935.9 | 1751.5 | 0.652 |
| LRHGE | 1307.5 | 1582.5 |  | 1518.6 | 1360.5 | 0.580 |
| Law's features |  |  |  |  |  |  |
| L1 | 430222.8 | 396033.4 |  | 280711.2 | 188960.5 | 0.243 |
| L2 | 85655.6 | 117052.5 |  | 36682.8 | 30557.2 | 0.182 |
| L3 | 15982.7 | 15796.4 |  | 8492.7 | 1482.8 | 0.142 |
| L4 | 108666.8 | 112217.6 |  | 57799.7 | 38663.6 | 0.155 |
| L5 | 21652.8 | 25234.7 |  | 11044.3 | 10426.3 | 0.190 |
| L6 | 15918.3 | 19827.0 |  | 8023.7 | 7422.1 | 0.210 |
| L7 | 8381.1 | 9064.8 |  | 4416.7 | 3807.9 | 0.174 |
| L8 | 37251.5 | 46942.5 |  | 18625.8 | 16764.7 | 0.210 |
| L9 | 43094.5 | 36937.3 |  | 26611.7 | 17031.3 | 0.170 |
| GLGM |  |  |  |  |  |  |
| MGR | 4.850 | 2.696 |  | 7.615 | 4.199 | 0.038* |
| VGR | 17957.5 | 10154.8 |  | 28800.9 | 15431.2 | 0.028* |
| skewness | 36.4 | 12.0 |  | 28.8 | 7.4 | 0.030* |
| kurtosis | 1532.4 | 1079.6 |  | 940.4 | 462.6 | 0.071 |

* = Significant differences are defined as *p* < 0.05

*Definition of abbreviations:* GLCM = gray level co-occurrence matrix; GLRL = gray-level run length; SRE = short-run emphasis; LRE = long-run emphasis; GLN = gray-level nonuniformity; RLN = run-length nonuniformity; RP = run percentage; LGRE = low gray-level run emphasis; HGRE = high gray-level run emphasis; SLRGE = short-run low gray-level emphasis; SLHGE = short-run high gray-level emphasis; LRLGE = long-run low gray-level emphasis; LRHGE = short-run high gray-level emphasis; GLGM = Gray level gradient matrix; MGR = mean gradients; VGR = a variance of gradients; SD = standard diviation.
